# Tendon Transfer in Hand Trauma: A Case Report

**DOI:** 10.5812/traumamon.7578

**Published:** 2013-01-15

**Authors:** Hossein Saremi, Ali Karbalaeikhani

**Affiliations:** 1 Orthopedics Department, Besat Hospital, Hamedan University of Medical Sciences, Hamedan, IR Iran; 2 Hand and Microsurgery Department, Emam Reza Hospital, Army University of Medical Sciences, Tehran, IR Iran

**Keywords:** Tendon Transfer, Amputation, Avulsion

## Abstract

In this report we describe our encounter of a case of hand trauma referred to our center presenting with incomplete left thumb amputation at metacarpal level with extensor pollicis longus (EPL) and flexor pollicis longus (FPL) tendon avulsion from the tenomuscular junction. After metacarpal bone fixation the ulnar digital artery was anastomosed and the digital nerves were coapted. Transfer of extensor indicis proprius (EIP) to EPL and side-to-side suturing of the FPL to the deep flexor tendon of the index finger were performed.

## 1. Case Report

The hand of a 24 year-old man injured during work in a factory referred to the emergency department of our hospital due to an incomplete left thumb amputation. The primary evaluation in the emergency department revealed an incomplete devascularized transmetacarpal amputation of the left thumb, attached to the hand with only a part of ulnodorsal skin of the thumb.

**Figure 1.**
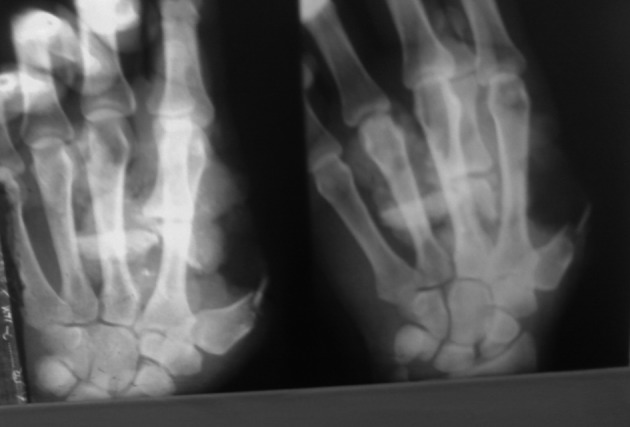
Radiography of Patient's Hand Reveals an Incompletely Amputated Thumb of the Left Hand

**Figure 2.**
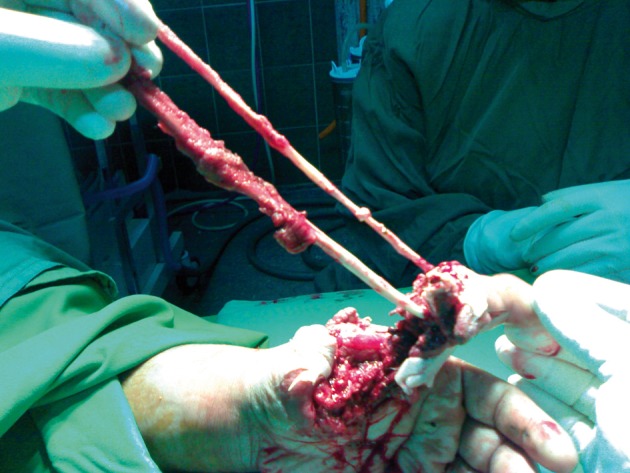
Intraoperative Photography Shows Tenomuscular Avulsion of EPL and FPL Tendons.

**Figure3.**
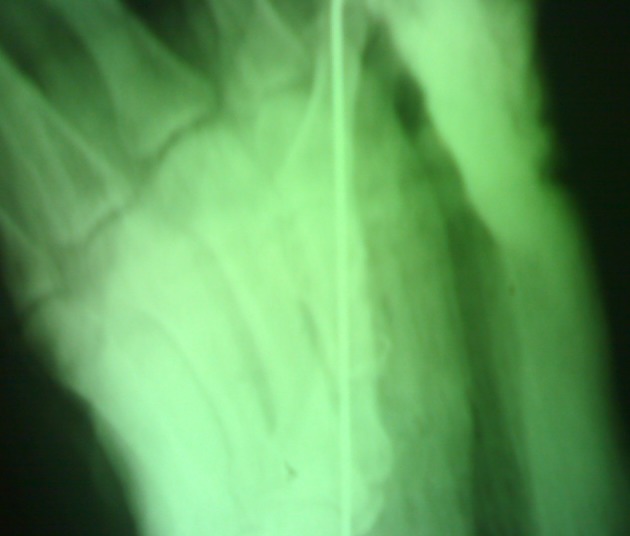
The First Metacarpal Fixation by Steel Wire and K Wire

Radiography demonstrated comminuted fracture of the metacarpal bone (Figure 1). Extrinsic tendons including flexor pollicis longus (FPL) and extensor pollicis longus (EPL) had been avulsed from tenomuscular junction (Figure 2). After the primary evaluation, the patient was transferred to the operation room. Under general anesthesia, the distal and proximal parts of the amputated thumb were evaluated. First metacarpal bone was shortened and fixed with K wires (Figure 3). Afterwards, the thumb was revascularized by the anastomosis of ulnar digital artery under operating microscope with 9/0 nylon suture and the digital nerves were coapted. Due to the skin bridge vein repair was not performed.

**Figure 4.**
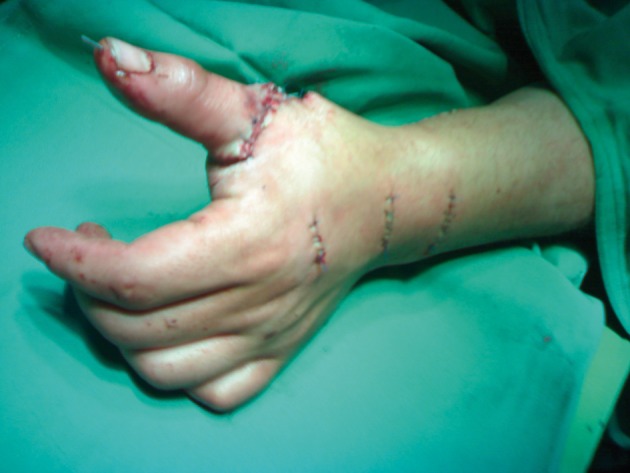
Incisions Repaired for EIP to EPL Transfer

In order to reconstruct the EPL tendon, the extensor indicis proprius (EIP) tendon was harvested through a short transverse incision just proximal to the metacarpophalangeal joint; the distal stump was then sutured end-to-side to the remaining extensor digitorum cummonis (EDC) tendon of the index finger to prevent extensor lag. The EIP was rerouted through second and third transverse incisions distal and proximal to the extensor retinaculum and was passed through the third extensor compartment of the avulsed EPL tendon. The rerouted EIP tendon was then sutured to the EPL tendon by the Pulvertaft weave technique (Figure 4).

**Figure 5.**
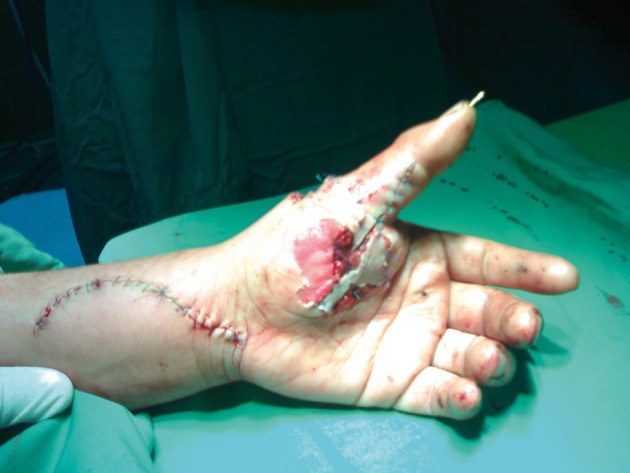
Incision for FPL to FDP Transfer

**Figure 6.**
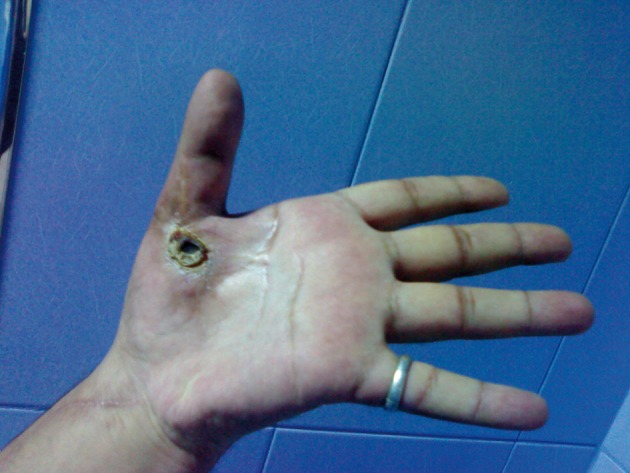
Left Hand of the Patient About one Month After Tendon Transfer With Complete Extension of Thumb With Local Thenar Area Necrosis

**Figure 7.**
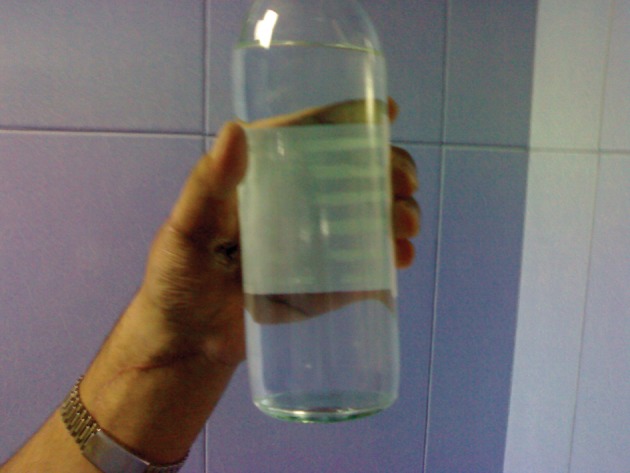
Grasping a Bottle by Patient

The avulsed FPL tendon was transferred to the anterior forearm under the transverse carpal ligament and was sutured side-to-side to the deep flexor tendon of the index finger (Figure 5). After the operation, necrosis of a small part of Thenar skin was noted, which later healed by secondary intention. At the short term follow-up, the patient was able to flex and extend the thumb and grasp, opposition and pinching were satisfactory (Figures 6 and 7). Tinel’s sign progression was detected in the digital nerve.

## 2. Discussion

The muscle tendon unit which is selected as the potential donor for transfer must be expendable and have strength and excursion similar to the paralyzed tendon. The index finger has two extensor muscles: EIP and EDC. One of them is enough for index finger extension. EIP has strength, excursion and direct line of pull similar to the EPL. The extensor indicis transfer is a simple and effective technique for reconstructing the thumb with low donor site morbidity. Spontaneous rupture of EPL tendon after distal radius fracture is a common complication and transfer of EIP tendon has been successfully used for the reconstruction of the thumb extension (1-3). If EIP is absent, Palmaris longus is another choice for reconstructing the thumb extension (4). Endoscopic EIP transfer has been used with single incision for EPL reconstruction in the distal radius fracture (5).

However, FPL tendon reconstruction has a different history. FPL reconstructions in emergency and elective settings are different procedures. Old rupture of the FPL is similar to congenital FPL deficiency. Congenitally absent and untreated ruptured FLP tendon is reconstructed in two stages. In the first stage, flexor pulleys are reconstructed and a silicone rod is inserted into the tendon sheath. In the second stage which is done in three to six months later, tendon of the flexor superficialis muscle of the ring finger is used as the motor source. There is no need for tendon graft in this procedure (6-9). Brachioradialis transfer of isolated FPL paralysis is used for thumb interphalageal joint flexion reconstruction with acceptable results in median nerve palsy (10).

Our patient who had referred to our center for emergency revascularization of incomplete thumb amputation had FPL and EIP disruption from the musculotendinous junction. His extrinsic muscles were not available for repair. To our knowledge, no report has mentioned the use of the flexor digitorum profundus (FDP) of the index finger for FPL reconstruction. Although this type of transfer results in concomitant movement of the thumb and index fingers, this was not a problem and our patient was satisfied with the result.

No secondary revision surgery was done for this patient. In long-term follow up the patient did not go back to his pre-injury occupation as a factory worker and is now working as a cab driver. In thumb replantation or revascularization procedures, the standard procedure of ring finger FDS transfer can be replaced by a side-to-side suturing of the FPL tendon to the FDP of the index finger hence preserving a functioning tendon in cases of failure.
